# How Has the Nationwide Public Health Emergency of the COVID-19 Pandemic Affected Older Chinese Adults' Health Literacy, Health Behaviors and Practices, and Social Connectedness? Qualitative Evidence From Urban China

**DOI:** 10.3389/fpubh.2021.774675

**Published:** 2022-03-10

**Authors:** Xiangnan Chai

**Affiliations:** Sociology Department, School of Social and Behavioral Sciences, Nanjing University, Nanjing, China

**Keywords:** COVID-19, older Chinese adults, health literacy, health behaviors and practices, social connectedness, semi-structural interviews

## Abstract

Older Chinese adults' daily lives have been affected significantly during the outbreak phase of the COVID-19 pandemic since January 2020. They were confronted with activity restrictions due to strict pandemic prevention. The older population also had to get accustomed to widely-used modern technologies in community management, such as health codes and WeChat groups. By late 2021, mainland China had reduced the prevalence of COVID-19, and people's daily lives had primarily returned to pre-pandemic normality. Under China's systematic health management during the pandemic, older Chinese adults' responses to this nationwide public health emergency may have influenced their health in the long run. However, it remains unclear what specific health changes or improvements have occurred. Such a void in the literature is worrying, given that older adults are at high health risks due to the pandemic which, might still be with humankind for a while. Thus, it is of necessity to explore and report their health changes after this official, large-scale health intervention. In this study, 17 adults aged 55 and above were recruited as interviewees. All interviewees reside in a community located in Q district, N city of the People's Republic of China. According to the findings, many interviewees now have better literacy in health risk prevention. Information and Communication Technologies (ICTs) play a significant role in getting access to health information. Specifically, television, WeChat chatting groups, and TikTok could be valuable information sources for older adults. As for the understanding and evaluation of health information, although older participants can distinguish COVID-19 rumors, they may sometimes feel confused about the underlying scientific logic. Regarding changes in health behaviors and practices, many older adults can integrate health information and knowledge into their daily lives. Additionally, although interviewees can keep important social connections, not all of them are familiar with using new ICTs, such as online chatting group, for social participation and engagement. The empirical evidence suggests that both the communities and the local governments can offer specific training programs to older residents for the sake of enhancing their health literacy, health behaviors and practices, and social connectedness during and after the pandemic.

## Background

Older adults are particularly vulnerable during the COVID-19 pandemic (1, 2). Even though the virus has affected all age groups, recent data show that the senior population has the highest mortality rates. In the initial stages of the pandemic, older adults' mortality rates in China, Western Europe, the United Kingdom, Canada, and the United States were eight to 62 times higher than adults 55 years and younger. Those aged 65 and above were found to have the highest mortality rate (3). Data show that, in the United States, COVID-19 deaths in 2020 totalled around 380,000, with approximately 80% aged 65 and above (4). Accordingly, Americans' life expectancy at age 65 drops by 0.75–0.94 years in the best and worst scenarios, respectively (5). These indicate glaring mortality disparities due to age. Besides fatalities, health challenges facing the aged have been well documented. Although there is some disagreement in the literature–a few experts found, for instance, that older adults were not negatively affected in terms of some specific health outcomes such as anxiety, stress, and depression [see (6, 7)]–plenty of other studies have demonstrated the opposite. A systematic research review found that mental health issues such as anxiety and depression commonly affected older adults during the COVID-19 lockdown (8). A certain proportion of older adults reported worse health behaviors compared to the pre-pandemic period (9). Other worrying global trends have been reported, including less exercising or inactivity (10, 11) and malnutritional behaviors (12–14). The COVID-19 crisis thus calls for urgent academic attention to underlying pathological and social factors contributing to the ways that older adults are confronted with possible health issues due to the pandemic. Additionally, in the Chinese context, existing studies have explored people's coping strategies toward pandemic risks, such as doing exercises at home and contacting community workers via online services (15, 16). However, more comprehensive research is needed to detect how older Chinese responded to the pandemic, and whether and how the pandemic has affected older Chinese's health literacy, health behaviors and practices, as well as social connectedness. Relevant explorations are crucial to understanding health situations facing older Chinese under COVID-19 and are thus essential to policymaking.

### Underlying Pathological Causes Leading to Older Adults' High Mortality Due to COVID-19

Existing pathological investigations have identified both immunosenescence and comorbidity as underlying reasons that can lead to older adults having higher likelihoods of being affected by COVID-19. Immunosenescence is defined as a functional decline in an individual's immune system due to aging (17). Napoli et al. (18) reported that the destructive effect of COVID-19 on older adults could be exacerbated by immunosenescence. Similarly, Sun et al. (19) researched older Chinese residing in Wuhan and identified that immunosenescence is a significant cause of their pneumonia. Accordingly, Brooke and Fahy (20) have proposed reversing immunosenescence as a means of COVID-19 prevention.

Comorbidity is another core mechanism that results in disproportionally high mortality within the older population (21). Sanyaolu et al. (22) reviewed related research and found that hypertension and diabetes aggravate the severity of COVID-19. Likewise, Sharma (23) modeled COVID-19 mortality among Americans and found that obesity and hypertension are both potential triggers accelerated by age. Additionally, a meta-analysis conducted by Wang and associates (24) supports the notion that comorbidity is an underlying cause of COVID-19 mortality; therefore, diagnosed patients should pay attention to comorbidities, including “hypertension, diabetes, chronic obstructive pulmonary disease, cardiovascular disease, and cerebrovascular disease” (p. 6056).

### Social Factors Contributing to Health Problems Facing Older Adults During the COVID-19 Pandemic

Besides pathological causes, social factors that contribute to older adults' health problems during the pandemic have also been well investigated. Although social distancing and quarantining have been implemented for pandemic prevention in many countries and regions, these required regulations may lead to social disconnectedness and isolation, especially for older adults (7, 25, 26). Under the pandemic context, existing research has shown that many older adults develop mental health issues as indicated by the co-occurrence of depressive symptoms (27, 28), affective disorder and suicide intentions (26), and other psychological concerns (29–31). Mata et al. (32) explored longitudinal data focusing on the daily lives of Germans during the pandemic; results indicate that health-promoting behaviors including less screen time, healthier food intake, and more exercising can improve people's mental health, including older adults.

Lack of financial support (33, 34) and health and medical resources (35) have been argued to be critical contributors to health risks and problems facing older adults during the pandemic. Many older adults have been economically challenged due to job market shrink. They are confronted with a reduced income or a shortage of other financial support. Scholars, therefore, warned of the urgency to focus on older adults living under disadvantaged economic conditions, such as poverty and food insecurity (29, 34). Unequal access to health resources due to geographic divides may also lead to disparities in health outcomes among the aged. For example, Henning-Smith (36) indicated that older adults residing in rural areas have fewer healthcare facilities compared to their urban counterparts. From a global perspective, scholars have also emphasized older adults living in less developed countries and regions because they lack necessary medical and life resources (37–39).

### Older Adults' Coping Strategies in Daily Health Practices Toward the Pandemic

Although older adults are at high risks of being negatively affected by the COVID-19 pandemic, mainly due to pathological and social factors (19, 33, 35), they may have the agency to develop resilience and coping strategies against the public health crisis (40). Chen and associates (15) interviewed 15 close contacts of diagnosed COVID-19 patients about their feelings and coping mechanisms at different stages of quarantine. Their interviewees actively used a couple of coping strategies, such as distraction strategies and keeping optimistic, throughout quarantine. Despite these crucial findings, interviewees recruited were aged between 18 and 60; there was no focus on the older population. Another important study was done by Yang and associates (16). The researchers interviewed 18 older adults aged 65 and above to investigate their experiences during the lockdown in 2020 in Wuhan. They found that older adults developed coping strategies, such as doing exercises at home and actively followed community management and government governance. Also, social support from families, communities, and the local government were crucial resources for older adults. Another remarkable finding is that older adults were able to use online chat groups or online platforms to order food and drugs and use online doctor services for health consultations. Despite these vital findings, further explorations are needed. Specifically, for instance, although the authors indicated that older adults used the internet to get updated pandemic information, no more details have been detected regarding the role of Information and Communication Technologies' (ICTs) use in this regard. Also, the research did not explore whether older adults were able to understand and evaluate key health information which is, however, important for making health decisions. Additionally, changes and improvements in older adults' health behaviors and practices after the first-wave pandemic remain unclear.

### The Chinese Context of Pandemic Governance

China has a clear timeline regarding the outbreak and development of the COVID-19 pandemic. As He, Shi and Liu (41) reported, China's domestic outbreak can be traced to December 2019 in Wuhan, Hubei Province. The human-to-human transmission was confirmed 11 days after the first death occurred on January 9, 2020; Wuhan was timely locked down three days after this confirmation. On April 8, 2020, China ended Wuhan's lockdown.

China has been exerting a “tough model” (42) targeting pandemic prevention at a macro level. Community mobilization during the pandemic was strictly regulated. Notably, China implemented rapid and concrete measures, including carrying out strict community mobilization registration, advocating mask-wearing and other effective preventive measures as crucial health information, building field hospitals (41), and promoting vaccine research (43). Some policies were implemented on the national scale during and after the Wuhan lockdown, such as using health QR codes for mobility identification^1^ and vaccine immunization. China's grid-style social management^2^ in every community assisted in community governance (41, 46, 47). Firm government control and concrete policy implementation have efficiently controlled the first-wave pandemic in China (48).

### The Current Study

The current study fills two literature gaps. First, under China's strict pandemic management, on the individual level, little is known about how the Chinese have waded through the pandemic and whether this public crisis has brought any changes to their health. Accordingly, this work uses the conceptual tool of “health literacy” for further explorations. In the 1970s, Simonds (49) put forward the concept of “health literacy,” highlighting the importance of health education as social policy for America's K-12 system. In 1998, the World Health Organization (WHO) defined health literacy as “the cognitive and social skills which determine the motivation and ability of individuals to gain access, understand and use information in ways which promote and maintain good health.” [See (50), p.264]. Health literacy can thus be seen as an individual's asset (51). Sørensen et al. (52) further put forward four dimensions of health literacy: access, understanding, appraisal, and application of health information. Getting access to health information and adjusting health behaviors have been argued as effective coping strategies during the pandemic quarantine (15, 16). Additionally, more comprehensive dimensions of older adults' access to health information, their understanding and evaluation of health information, as well as using health information as primarily indicated by any changes and improvements in health behaviors, yet remain unknown. It is also unclear how the pandemic has affected older adults' social connectedness. However, keeping socially connected is crucial for older adults' health maintenance through obtaining necessary health information and achieving a wide range of support from families, friends, and neighbors (53). Hence, the current study aims to contribute to related literature by addressing the research concerns of whether and how the COVID-19 pandemic has affected older adults' health literacy, health behaviors and practices, and their social connectedness. The roles of national and community policies have also been taken into account as macro- and meso-level contexts.

The other literature gap is that not many academic efforts focusing on the Chinese context are qualitative. Two of the very few qualitative studies exploring Chinese people's involvement, experiences, and coping strategies under the pandemic were conducted by Chen and associates (15) and Yang and colleagues (16). This dearth in the literature results in little known about older Chinese adults' actual life experiences and feelings related to the pandemic. Accordingly, this work uses semi-structured interviews to detect older adults' lives during the pandemic through a qualitative lens.

## Methods

### Data Collection

This study uses semi-structural interviews. The research team collected data in a demolition and resettlement community located in Q District, N city, the People's Republic of China. The author held training sessions for six research assistants who helped collect data. The interviews were done in April 2021. In total, 17 adults who have lived in the community for years and aged 55 and above were recruited with the assistance of a community leader.

The interview outline includes six sections. The first and last sections have questions on interviewees' essential demographic and socioeconomic background, including age, gender, educational attainment, employment, and living arrangements. Also, as previous studies explored, older adults with health problems may have been affected more severely compared to their healthy counterparts (22, 24). Therefore, the second section is about health status, including interviewees' self-rated health and diagnosed chronic diseases. In the third section, interviewees were asked about their perceptions of this pandemic, including their feelings about daily governance by communities and governments. In the fourth and fifth sections, interviewees were asked about their health literacy, including access to essential health information, how they understand and evaluate pandemic rumors, and their application of health information. Interviewees' health behaviors and practices before and after the first-wave pandemic, including daily exercising, smoking, drinking, handwashing, mask-wearing, and ventilation, were considered important aspects of applying health information. Interviewers also asked about whether and how older adults' social connections have changed since maintaining social connectedness helps the aged in health management (54).

Although the interview outline is somewhat structured, interviewees were encouraged to recall and tell their authentic experiences and feelings. Interview questions consider participants' demographic and socioeconomic backgrounds, individual experiences, and community management; this aims to provide relatively panoramic life experiences of older participants under the pandemic and ensure the validity of research findings (55). All participants were interviewed based on the same interview guide.

### Data Analysis

The author rigorously conducted data analysis by taking the following steps. First, all interview records were transcribed into text files. The author then read transcripts several times to get familiar with the data. A Microsoft Word keyword search was used search during the reading process. Notably, because the accuracy of automatic transcription was affected by dialects, the author carefully compared interview audios and transcripts to avoid any possible misinterpretations. Because this study has specific research topics, the interview outline is to a degree structured; thus, interview transcripts can be categorized into themes regarding older adults' health literacy, health behaviors and practices, and their social connections. Further, the author reread transcripts and then used a word search and MAXQDA software program to generate subthemes through coding line by line, including “getting access to health information using traditional ICTs,” “getting access to health information using new ICTs,” “health practices that are relatively easy to develop,” “health behaviors that are difficult to change,” and “using new ICTs to maintain social connections.” These subthemes are closely related to the research concerns and were widely reported by the interviewees so that they can sufficiently cover the qualitative data that have been collected. Finally, a research assistant separately read all transcripts and checked themes and subthemes generated by the author and interviewees' quotes that are featured in this paper. The author and the assistant reached a consensus after discussions.

### Research Ethics

This study highlights the application of research ethics. The Psychology Department at the School of Social and Behavioral Sciences of Nanjing University gave ethical approval for the current research, and the approval code is NJUPSY202106001. The interview began with clarifying interviewees' rights, including quitting the interview whenever they wanted. All interviews and discussions are reported anonymously to protect interviewees' privacy and other personal interests. Also, the author paid all interviewees for their participation and contributions.

## Results

Table 1 presents the demographic characteristics and self-rated health of the interviewees. In the interviews, 17 older adults aged between 55 and 74 years were recruited. Their average age is 63.59 years, and most interviewees (64.71%) are between 60 and 69. Nine of the interviewees are male. As for their educational attainment, three (17.65%) are below middle school, and the rest have middle school or high school degrees. Interviewees' educational backgrounds may help facilitate their use of ICTs, such as televisions and the internet, to access helpful health information during the pandemic. More than half (58.82%) of the interviewees are retired; two of the rest are doing part-time jobs, and five are formally employed. All of the interviewees live with their partners, one of whom live with her adult children and grandchildren. None of them live alone. Regarding self-rated health, most reported that they were currently in good health status (64.71%), and none of them reported poor health. As stated, 88.24% of the interviewees have chronic diseases. But as rated and reported by the interviewees, these diseases do not heavily affect their daily lives. Appendix 1 presents detailed information on each interviewee's demographic background and self-rated health.

**Table 1:** Demographic characteristics, self-rated health, and diagnosed chronic diseases of interviewees aged 55 and above, *N* = 17.

	**N**	**Mean or %**
**Age**		63.59 years
Below 60	4	23.53%
60–69 years	11	64.71%
70 years and above	2	11.76%
**Gender**		
Female	8	47.06%
Male	9	52.94%
**Educational attainment**		
Below middle school	3	17.65%
Middle school	6	35.29%
High School	8	47.06%
**Employment**		
Retired	10	58.82%
Part-time	2	11.76%
Employed	5	29.41%
**Living arrangements**		
Living with partners	16	94.12%
Living with partners, children, and grandchildren	1	5.88%
**Self-rated health**		
Average	1	5.88%
Good	11	64.71%
Very good to excellent	5	29.41%
**Diagnosed chronic diseases** ^a^		
Yes	15	88.24%
No	2	11.76%

a *Diagnosed chronic diseases, such as hypertension, cardiac diseases, hyperglycemia, and osteoporosis, are common among older Chinese people (56)*.

This study mainly focuses on older adults' health literacy, health behaviors and practices, and social connectedness. Health literacy includes their access to, and understanding, evaluation, and use of health information. The use of health information is reflected by changes and improvements in health behaviors and practices in daily life. Social connections are also important sources for gaining essential health support, such as health information and emotional support. To preface research findings, results first indicate that ICTs have become an efficient method for their access to health information. Also, interviewees were able to evaluate pandemic rumors as false information, but they may not understand the underpinning scientific logic. Notably, some older participants strategically applied related information to daily life practices, as indicated by some clear improvements in health behaviors and practices. But some participants showed no differences in their health literacy, health behaviors, and health practices after the major wave of the pandemic compared to their pre-pandemic statuses. Moreover, interviewees maintained social connectedness to receive emotional and social support and kept themselves updated on health information and pandemic news.

### The Access to Crucial Health Information: The Role of Traditional and New ICTs

Access to key health information is one significant dimension of health literacy (50–52). The research results show that ICTs play a crucial role in helping older participants obtain health information, improving their health literacy. Traditional media, such as broadcast and television, still plays a crucial role in information access. “*We got to know [pandemic] information through radio broadcast and community advocation. We wore masks when we went outside, and we didn't go to crowded places*.” (N5, male). Compared to the radio broadcast, television is more widely reported as a way of getting health information. “*I [created] my WeChat [account] recently, but I don't know how to use it. … I watched television and got to know the pandemic information*.” (N1, female). Likewise, as another interviewee said, “*We got to know health information via television. For instance, television channels often broadcasted pandemic news, so we were able to know what was going on and what to do*.” (N3, female). This interviewee trusts China Central Television (CCTV), China's state television channel, and the provincial television channels as primary information sources. Similarly, official television news represents authority to some older adults who do not trust new media platforms but wish to know factual pandemic news. For example, a male interview (N16) said, “*Television is the best way to get access to health information. I watch television every day. Cellphone [news] is another way, but it is sometimes correct and sometimes incorrect*. *[But] news on television channels, CCTV or local official channels must be correct*.”

The internet and some new communication methods, such as WeChat instant chatting group, have emerged as another powerful method for many but not all older adults. “*I watch online news via the channel of Tou'tiao (Headline, a Chinese e-news platform) every day. I also got to know pandemic information via community WeChat groups*.” (N14, female). Also, according to interview transcripts, WeChat instant chatting group is widely accepted and welcomed by many interviewees. The main reason is that, in this way, older residents can get to know about pandemic news and community policies concerning pandemic governance in a timely manner. Some older adults have also used some other new media platforms, such as TikTok, showing a consistency with prior research, which indicates that many older Chinese have begun to use new ICTs (57, 58). An interviewee said that she has gotten used to learning body mechanics and accessing pandemic prevention information on TikTok (N6, female). Similarly, another interviewee (N3, female) reported that she sometimes watched short videos regarding pandemic prevention (e.g., mask-wearing and handwashing related) on TikTok. Moreover, one interviewee (N17, male) reported that, besides television channels and WeChat groups, he has also begun using smart speakers to get health information. As the interviewee said, “*I ask the smart speaker to give me the current pandemic situation, and it can respond to me immediately.”*

### Understanding and Evaluating Health Information: How Did Older Participants Treat COVID-19 Rumors?

Two interview questions about prevalent COVID-19 rumors were included in the outline to detect how older adults understand and evaluate health information. The first question is, “whether you have heard the news that shuang'huang'lian oral liquid (a traditional Chinese patent medicine^3^) can prevent or cure COVID-19, and whether you believe it or not.” Some interviewees said that they do not trust such statements, “*[Although] I've heard about this statement, I think it is not reliable. I don't trust it*.” (N13, male). Some expressed that scientific or medical authorities should be the only trustable source when understanding and evaluating health information. An interviewee said that “*No, I don't trust this [rumor]. If you got affected, you should go to the hospital*.” (N14, female). Likewise, a male interviewee (N11) said that “*I don't think it [the rumor] is reliable. It is probably a type of advertisement. For example, Hong'mao medicinal wine (note: a type of wine and a Chinese healthcare product). … I heard that my daughter-in-law and aunt who had used the medicinal wine said it is not that useful. It is not as effective as what they said [in the advertisement]. Therefore, I don't think I would believe such statements. You should keep optimistic to maintain your own health*.”

Notably, to some interviewees, evaluating health information depends on whether the information comes from the government. For example, an interviewee demonstrated that he only trusted what the government said about the pandemic, “*I kind of heard this [statement]. We generally don't trust it. It's better to follow local pandemic reality to decide which [piece of information] to trust or distrust. There are so many rumors outside, but we normally don't believe them. The government can [and would] transfer [pandemic] information to ‘Red Posthouse' (note: a newly emerged type of activity center for community residents and Party members) through Party branches to [further transfer pandemic information] to community residents*.” (N5, male).

The interview outline includes another question, “whether you have heard drinking alcohol can prevent or cure COVID-19, and whether you believe it or not,” because drinking is common among older Chinese adults. Similarly, although some participants or their partners have developed the alcohol drinking habit for years, most do not believe that drinking would cure COVID-19. As an interviewee (N7, male) explained, “*I drink a little bit of wine every day…but it is just a personal habit. I don't trust that drinking could prevent COVID-19*.” Another male interviewee (N11) said, “*I haven't heard statements about drinking, but I've heard similar things about smoking. I didn't get to know the information from the television news or newspapers but from neighbors. They said that those infected patients are nonsmokers; the likelihood of smokers getting infected is lower. I think such a statement is without scientific evidence. Infection is due to an individual's low immune system*.”

To briefly conclude, according to the transcripts, most participants are able to evaluate and distinguish these two pandemic-related rumors correctly. As some expressed, these rumors do not make sense, and they trust health information offered by medical doctors and advocated by the government. However, older adults may find it difficult to clearly understand the underlying scientific logic why the Chinese patent drug or alcohol could not prevent or cure COVID-19. For example, an interviewee said, “*I don't take [these] medicines. I don't understand it, and I also don't need [shuang'huang'lian oral liquid]. I have never heard of this information; I am healthy, so my family never has these things. My husband is healthy too. We don't really need it*.” (N9, female). Another interviewee (N2, female) said that “*My cousin relayed the information regarding shuang'huang'lian oral liquid to our WeChat group. I don't trust it could cure COVID-19. But I know that another drug named tu'mei'su (oxytetracycline) could be useful. Tu'mei'su was widely accepted in rural areas during my childhood. I heard that it would work, but I am not sure*.”

### The Application of Health Information: Observed Modes of Changes in Health Behaviors and Practices

The application of health information is another critical dimension of health literacy, which can be indicated by changes in people's daily health practices and behaviors (52). A couple of modes have been observed based on data analysis.

For many interviewees, the pandemic has brought some changes in their daily lives in that they have paid more attention to daily health practices and have developed healthier behaviors. For example, many reported that “thanks to” the COVID-19 crisis, they got to know the efficiency of hand washing and mask-wearing, which WHO highly recommends as efficient pandemic preventive methods (60). “*I learned the seven-stage hands washing method during the pandemic*.” (N2, female). “*Now, I know the importance of improving immunity. I am aware of the irreplaceable importance of my health. I've developed habits of wearing masks when necessary (such as for cough prevention) and washing hands*.” (N16, male). Similarly, after the first wave of the COVID-19 pandemic, some interviewees reported that they have been exercising more often since last January. A female interviewee said that, “*I do daily exercise more often after the [first-wave] pandemic than before. I didn't care this much about exercising before the pandemic*.” (N8, female). Likewise, another interviewee said, “*I now pay more attention to exercising for to health [improvement]. Some people [at my place of work] do fitness. I also do fitness sometimes. I participate in jogging activities when available*.” (N7, male).

Some interviewees have practiced these beneficial health behaviors for a long time, so the pandemic rarely affects them. For instance, one interviewee is a public health expert, so she knows much health knowledge and emphasizes family health. The pandemic thus has a minimal impact on her daily health practices (N3, female). Similarly, another participant said, “*For years I do exercise 3 hours per day [on average], basically walking in the park: 1 hour in the morning; 1 hour in the afternoon, and 1 hour in the night*.” (N5, male). A female interviewee (N6) reported paying a lot of attention to maintaining health and usually walked in the community part 1 hour per day. The pandemic did not stop her from doing this exercise. From this perspective, the findings align with prior studies that older Chinese pay much attention to health management or improvement through physical activities (61, 62).

Even though the pandemic has brought positive changes in older adults' health practices and behaviors, some more destructive health behaviors are not easy to change even under the pressure of COVID-19. For example, some older men have been smoking and drinking for a long time, so they feel that making positive changes will not make a big difference. A female interviewee said that “*The pandemic didn't change my husband's smoking habit, as he told me that quitting smoking for him is impossible*.” (N1). Similarly, another interviewee reported that her husband “*Continues to drink and smoke. But he has the self-awareness not to go to crowded places such as parks*.” (N2, female). As a male interviewee (N16) demonstrated, he has now reduced smoking frequency compared to his pre-pandemic volume, but he continues to smoke nevertheless.

### Keeping Socially Connected as a Method for Health Maintenance

This research lastly investigated older adults' social connectedness during the pandemic. No evidence suggests that interviewees were confronted with disconnectedness or isolation issues during this public health crisis. This may be due partially to the fact that all interviewees live with their partners, and many live close to their children and grandchildren. Many interviewees took on daily caregiving tasks if their grandchildren were young; such intergenerational interactions enrich their daily lives, which may help them to avoid social isolation and loneliness.

Results indicate that ICTs also helped interviewees to maintain connection during the pandemic. This is in line with existing studies, which document that ICTs have become common among older Chinese for social connections (57, 63). As reported, all interviewees have used feature phones or smartphones to stay in touch with siblings, other relatives, and friends. For example, one interviewee (N5, male) said that “*Basically we called them (note: his daughter's family) and told them not to come back [during the pandemic] because it was unsafe for them on the way back…We called them every once for a while to ask how things were going*.” Additionally, some (but not all) interviewees are familiar with the most widely-used Chinese smartphone apps, such as WeChat. These new media platforms provide older adults with a convenient online community where maintaining timely connections is attainable. “*I truly like using WeChat. I often talk with friends and relatives using WeChat instant video calls*.” (N2, female). Similarly, “*WeChat typing or video chatting makes [communication] more convenient than before. I think it is truly good*.” (N15, male). Hence, using new ICTs during the quarantine may have prevented older adults from being forced into social disconnectedness or isolation, which helps them avoid being negatively affected in terms of their health. Older adults can interact with each other online during the pandemic, reducing possible risks due to face-to-face interactions. They can get timely pandemic news and health information from their social connections. For example, WeChat groups have been used widely to share news efficiently. “*Householders are in the WeChat group. If community workers need to announce anything, they do that through WeChat group chatting*.” (N3, female). Likewise, an interviewee said, “*We have a big WeChat group with [more than] three hundred community residents. Our neighbors remind each other about safety and security during the pandemic. We also relayed [pandemic news and community announcements] to the chatting group*.” (N4, male).

## Discussion

The current study is based on a specific context in which older adults worldwide face health risks due to the COVID-19 pandemic (3, 38). This study focuses on health literacy, health behaviors and practices, and social connectedness of older Chinese adults due to the COVID-19 pandemic. It is among the limited qualitative endeavors to explore pandemic experiences among older Chinese specifically. Findings show effective self-adjustment of older adults to avoid possible physical and mental problems. Many older Chinese can maintain or improve their health during and after the pandemic with the efficient assistance of scientific and systematic community governance and wide use of ICTs.

Findings first shed light on the remarkable role of ICTs–both traditional and new ones–in access to health information among older Chinese; however, new ICTs deserve closer attention because not all older participants are familiar with them. Television is the primary type of traditional ICTs that the interviewees said that they watched televisions for gaining health information during the pandemic. New ICTs include the internet, WeChat chatting groups, TikTok, and so forth. Worth noting is that some interviewees know little about these new digital technologies. For example, an interviewee (N10, female) said, “*What does TikTok mean? We older people don't quite understand these [apps]. It's probably because we are old and unable to keep pace with these [new things]*.” Moreover, not all older adults have positive experiences of using new ICTs, and that is also noteworthy. Some interviewees (N = 4) do not have smartphones; their in-use phones have limited functions. They also reported inconvenient experiences due to the prevailing application of digital technologies in pandemic prevention. An example is that when these older adults were required to use new ICTs for community entrance, they felt inexperienced and thus depressed. “*Those technologies don't work very conveniently for older people. I feel a bit annoyed about the health QR code, but it works well for young people and the entire community governance*.” (N11, male). Hence, one related policy suggestion is that communities where older adults dwell should set age-friendly training programs on ICT use to increase access to key health information and social engagement and involvement (25).

Findings regarding observed modes of conduct indicate the complexity of changes in health behaviors and practices among older participants. It is not difficult for older participants to improve some health behaviors and practices, such as exercising, washing hands, and mask-wearing. These changes are primarily driven and impacted by health-promoting community advocation and changes in collective health patterns. But some longstanding, unhealthy behaviors, such as smoking, are not as easy to quit. One interviewee reported that he has been working on quitting smoking and drinking (N5, male), but the underlying motivation is not related to the pandemic. A policy suggestion in this vein is that community advocation should take into consideration both health practices that are relatively easy to develop and some long-lasting destructive behaviors that are prevalent among older Chinese adults.

Regarding older adults' understanding and their evaluation of health information (pandemic rumors), findings reveal an inconsistency between interviewees' understandings and evaluations of pandemic health information. Older adults' evaluations are primarily affected by news from authoritative sources and their own life experiences, but they may not completely understand the scientific mechanisms behind them. Such inconsistency may lead to incorrect evaluations of more puzzling pandemic rumors, making it essential for older adults' families and communities to offer necessary consulting services on this subject.

Findings also indicate that interviewees lived with families and could maintain social connectedness during the pandemic. Older adults can and do avoid isolation and loneliness and reduce health anxiety during the pandemic through receiving necessary emotional, financial, or other support from important social connections with families, friends, and neighbors (28, 64, 65). Moreover, these findings are consistent with existing ones that demonstrate that, with the assistance of ICTs, older adults can get timely health information and other health-promoting resources from robust connections (16, 54).

Further, although many efforts have been used to detect mechanisms linking older age and negative health consequences, the fact that some older adults have their agency, autonomy, and resilience for pandemic prevention and self-protection should not be ignored. As one interviewee said, “*I am not afraid of this pandemic at all. I had never developed the sense of fear. I even wished to be a volunteer when the virus hit Wuhan. I volunteered for community affairs last year*.” (N2, female). This interviewee is 61 years old and can be seen as a ‘young-old' that can be defined as older adults aged below 70 (66, 67). She now works as a caregiver for the ‘middle-old' and ‘oldest-old' in a community institution. She is self-perceived as a “*retired person*” but who wishes to continue to “*make contributions [to the society]*.” Some other interviewees also worked as community volunteers during the first-wave pandemic. “*We took temperatures for [the community residents]. We worked as volunteers during the pandemic*.” (N4, male).

Policy implementations regarding pandemic governance at the macro- and meso-levels also have exerted influences on older Chinese adults' health. When China underwent the first-wave of COVID-19, both the national and local governments took strict measures for pandemic prevention (41, 42, 46, 47). China's solid and effective governance and considerate community management aim to create a secure and friendly living environment for older adults. The interviewees hold positive attitudes toward the strict community governance during the first wave of the pandemic. Interview transcripts of this study show that the community applied multiple means, including measuring temperatures, personal information registration and management, and public sphere disinfection. During the crisis, community leaders, workers, and volunteers provided services for all dwelling residents. As some interviewees reflected, “*[Our] community leader, other [community] workers, and volunteers worked hard for the pandemic prevention. You could see them everywhere, especially when there were public health emergencies*.” (N11, male). Likewise, “*The community workers took care of every aspect of our community well during the pandemic*.” (N12, male). China's community management and service systems have been designed to cover all dwelling residents (41). Therefore, health inequalities due to age gaps may have been reduced due to these beneficial regulations based on national and community-level policies. Improvements in collective health patterns may also have positively impacted older Chinese.

### Limitations and Future Studies

This study is not without limitations. One limitation is that research findings cannot represent the older Chinese population at a national level. Interviewees are from urban areas in East China. Most interviewees share relatively similar demographic and socioeconomic backgrounds. None of them live in poverty or live alone, and none of them have urgent financial or healthcare needs. However, disparities in household income, organization membership, participation in city activities, and social capital have been shown to be contributors that lead to stratified health outcomes among older Chinese (27, 68). Thus, findings of this study can only laterally reflect pandemic experiences of older Chinese adults. It has been argued that understanding and meeting the care needs of older adults is essential for policymaking (69, 70). I thus call for more qualitative explorations and large-scale quantitative measures to help give us a panoramic picture regarding changes in health literacy, health behaviors and practices, and social connections among older adults due to the COVID-19 pandemic, and underlying reasons contributing to possible health inequalities that exist among the aged.

Another limitation is that this study did not pay close attention to the COVID-19 vaccine, which has been promoted nationwide in China. “More than 2.12 billion doses of COVID-19 vaccines had been administered” as of September 9, 2021 (71), benefiting approximately 970 million Chinese as of September 6, 2021, or about 77% of the country's total population (72). Hence, the next step is to investigate how the vaccine has affected older Chinese's daily health practices and health outcomes. Vaccinations may have long-term physiological and sociological meaning, especially considering that the pandemic has become recurrent as a consequence of new variants of COVID-19.

The third limitation is that this study uses retrospective data. Some interview questions on health change before and after the first-wave pandemic are retrospective, which may decrease the accuracy of the data. Timely data collection and explorations are thus required under new pandemic situations. A most recent pandemic condition is that new COVID-19 variants, such as Delta and Omicron, have meant that the local epidemic now rebounds back and forth. For example, N city had experienced a new city-level wave of the Delta variant pandemic in July and August 2021. Now, Omicron has emerged as a predominant variant in some countries and regions (73). As a response, China now implements regular pandemic prevention and control policies (74). While this policy benefits public disease prevention, it may bring new challenges and health risks facing older adults. For example, an individual's updated health QR code is required for public area entrance, which may become an obstacle and a new psychological stressor to the life of those older adults who are not familiar with digital technologies. These new pandemic facts therefore warrant further timely explorations into older Chinese's daily lives to understand their health challenges and needs, such as the need for expedient psychological services due to self-stigmatization and victimization under the pandemic (75–78).

The final limitation is due to the fact that interviews were conducted within a limited time. The temporary and formal interactions may have made it difficult for the author to profoundly capture more dimensions, details, and realities regarding older adults' daily health practices and social engagement. Considering that older Chinese have developed and practiced own life logic over their life courses, longitudinal and more comprehensive fieldworks through the author's participation and observation are thus further needed.

## Conclusion

This study uses semi-structural interviews to collect qualitative data among older Chinese residents living in N city, an east-coast city with a developed economy. Findings show that interviewees were able to handle the COVID-19 public health crisis strategically. Specifically, older Chinese adults were able to maintain or improve health literacy as well as health behaviors and practices to stay healthy. Also, older Chinese have autonomy and agency to keep themselves socially connected, thus avoiding loneliness and isolation and gaining health information and news. Remarkably, ICTs play a significant role in obtaining health information and keeping themselves connected with families, friends, and the community they dwell in. ICTs include traditional ones, such as television, and newly emerged technologies, such as WeChat instant chatting group and TikTok. But not all older adults were familiar with new technologies, especially those who do not have smartphones. This digital gap may have created obstacles for this segment of older Chinese because new ICTs have been widely used in pandemic management and governance. There also exists a gap between older adults' understanding and evaluation of health information. Specifically, although older adults were able to evaluate pandemic rumors correctly, they may not wholly understand underlying scientific logic. Policy recommendations were developed based on these findings.

## Data Availability Statement

The original contributions presented in the study are included in the article/supplementary material, further inquiries can be directed to the corresponding author.

## Ethics Statement

The studies involving human participants were reviewed and approved by the Psychology Department at the School of Social and Behavioral Sciences of Nanjing University (NJUPSY202106001). The patients/participants provided their oral informed consent to participate in this study.

## Author Contributions

XC proposed and developed the research idea, organized and participated in data collection, conducted data analysis, and wrote this manuscript.

## Funding

The author acknowledges funding from the project, “Study of health awareness and health behaviors of older adults in Jiangsu province in the post-pandemic Era (20SHC003),” the Social Science Fund of Jiangsu Province, Jiangsu Planning Office of Philosophy and Social Science.

## Conflict of Interest

The author declares that the research was conducted in the absence of any commercial or financial relationships that could be construed as a potential conflict of interest.

## Publisher's Note

All claims expressed in this article are solely those of the authors and do not necessarily represent those of their affiliated organizations, or those of the publisher, the editors and the reviewers. Any product that may be evaluated in this article, or claim that may be made by its manufacturer, is not guaranteed or endorsed by the publisher.

## Footnotes

^1^Health QR codes are used for identifying people's daily routine and health status. A colored code is attached to widely-used apps, such as Alipay. Green refers to (1) not a close contact, (2) no travelling to medium or high-risk areas, (3) no COVID-19 symptoms, and thus (4) health.

^2^Grid-style social management is a way of social management in China. According to Cai (44), an administrative district is normally divided into several segments (grids), and each segment is assigned to a “designated person” (para. 2), aka grid manager, whose working contents and responsibilities mainly concentrate on collecting information of their assigned segment and regularly submitting information to the “next higher level of authority.” (para. 3). Grid-style social management works efficiently in disease prevention and control during the pandemic (45).

^3^Existing research indicates that traditional Chinese medicines may have “good therapeutic efficacy in the treatment of COVID-19” [see (59), Abstract, para.4]. Nonetheless, that shuang'huang'lian oral liquid can prevent or cure COVID-19 is a pandemic rumor.

## Abbreviations

CCTV: China Central Television
ICTs: Information and Communication Technologies
NHC: National Health Commission of the People's Republic of China
WHO: World Health Organization.

## Appendix

**Appendix 1:** Demographic background and self-rated health of each interviewee, *N* = 17.

	**Gender**	**Age**	**Education**	**Employment**	**Living arrangements**	**SRH*^a^***
N1	Female	68	Primary school incompletion	Retired	Living with partner, son, daughter-in-law, and grandchildren	Good
N2	Female	61	High school	Employed	Living with partner, close to son	Average
N3	Female	74	Middle school	Part-time	Living with partner, close to children	Very good
N4	Male	63	Middle school	Retired	Living with partner, close to children	Excellent
N5	Male	68	Primary school	Retired	Living with partner, close to son	Excellent
N6	Female	58	Middle school	Retired	Living with partner, close to daughter	Good
N7	Male	55	Middle school	Employed	Living with partner	Very good
N8	Female	62	High school	Retired	Living with partner, close to daughter	Good
N9	Female	63	High school	Part-time	Living with partner	Good
N10	Female	66	Middle school	Retired	Living with partner, close to son	Good
N11	Male	66	High school	Employed	Living with partner	Good
N12	Male	56	High school	Employed	Living with partner	Good
N13	Male	70	Middle school incompletion	Retired	Living with partner	Good
N14	Female	64	High school	Retired	Living with partner	Good
N15	Male	57	Middle school	Employed	Living with partner	Very good
N16	Male	65	High school	Retired	Living with partner	Good
N17	Male	65	High school	Retired	Living with partner, close to children	Good

a *SRH refers to respondents' self-rated health. Although most of the interviewees have chronic diseases, their self-rated health statuses are in general at a relatively good level or above. This may indicate that the chronic diseases affect older interviewees' daily lives only slightly or remotely*.
